# Bystander activation across a TAD boundary supports a cohesin-dependent transcription cluster model for enhancer function

**DOI:** 10.1101/gad.352648.125

**Published:** 2025-09-01

**Authors:** Iain Williamson, Katy A. Graham, Matthew Woolf, Hannes Becher, Robert E. Hill, Wendy A. Bickmore, Laura A. Lettice

**Affiliations:** 1 MRC Human Genetics Unit, Institute of Genetics and Cancer, University of Edinburgh, Edinburgh EH4 2XU, United Kingdom;; 2 The Roslin Institute, University of Edinburgh, Midlothian EH25 9RG, United Kingdom

**Keywords:** 3D genome, cohesin, CTCF, DNA-FISH, loop extrusion, RNA-FISH, sonic hedgehog, transcriptional hubs

## Abstract

Mammalian enhancers can regulate genes over large genomic distances, often skipping over other genes. Despite this, precise developmental regulation suggests that mechanisms exist to ensure enhancers only activate their correct targets. Sculpting of three-dimensional chromosome organization through cohesin-dependent loop extrusion is thought to be important for facilitating and constraining enhancer action. The boundaries of topologically associating domains (TADs) are thought to prevent enhancers acting on genes in adjacent TADs. However, there are examples where enhancers appear to act across TAD boundaries, but it has remained unclear whether a single enhancer can simultaneously activate genes in different TADs. Here we show that some Shh enhancers can activate transcription concurrently not only at *Shh* but also at *Mnx1* located in an adjacent TAD. This occurs in the context of a chromatin conformation maintaining genes and enhancers in close proximity and is influenced by cohesin. To our knowledge, this is the first report of two endogenous mammalian genes transcribed concurrently under the control of the same enhancer and across a TAD boundary. These findings have implications for understanding the design rules of gene regulatory landscapes and are consistent with a transcription cluster model of enhancer–promoter communication.

## Abstract (precis)

In this study, Williamson et al. show that an Shh enhancer (ZRS) concurrently activates transcription of Shh (within its own topologically associating domain [TAD]) and Mnx1 (present in an adjacent TAD) by the cohesin-mediated regulation of enhancer–promoter proximity across the TAD boundary. Their work supports a model of enhancer function in which enhancers can promote the formation of transcription hubs that activate the transcription of genes within their sphere of influence.

How enhancers can act over very long genomic distances is still not understood. In mammals, the correlation between the regulatory landscapes of developmental genes, such as that of *Shh*, and topologically associating domains (TADs) suggests a role for 3D genome organization, particularly through cohesin-dependent loop extrusion (Symmons et al. 2014, 2016; Andrey and Mundlos 2017). Indeed, cohesin is required for enhancers to activate target genes located far away in the linear genome (Calderon et al. 2022; Kane et al. 2022; Rinzema et al. 2022). A similar correlation exists between TADs, their boundaries, and the ability of enhancers to activate reporter genes at various genomic positions, suggesting that TADs constrain enhancer activity (Symmons et al. 2014). However, there are examples in mammalian genomes where enhancers appear to be able to act across TAD boundaries (Beccari et al. 2021; Hung et al. 2024), suggesting that TAD boundaries are not completely impervious to regulatory information.

In vitro, CTCF can impede cohesin movement along DNA (Davidson et al. 2023), and in vivo, cohesin accumulates at oriented CTCF sites at TAD boundaries (Rao et al. 2014). However, CTCF depletion has only a small acute effect on transcription in cell culture (Nora et al. 2017; Hsieh et al. 2022). Similarly, experiments to test the in vivo effects of deleting CTCF sites at TAD boundaries have not given clear insight into the role of TAD boundaries on enhancer function. Deletions that encompass CTCFs sites 1 Mb from *PITX2* in families with a cardiac disorder result in TAD fusion and dysregulation of *PITX2* in heart tissues (Baudic et al. 2024). In mice, a deletion encompassing multiple CTCF sites at the TAD boundary between the Fgf3/Fgf4/Fgf15 locus and *Ano1* results in severe craniofacial abnormalities, perinatal lethality, and ectopic expression of *Fgf* genes in *Ano1*-expressing regions of the brain. This could be linked with loss of a single CTCF site (Chakraborty et al. 2024). Disruption of this same TAD boundary leads to ectopic *FGF3* activation driven by an *ANO1* enhancer in a human cancer cell line (Kim et al. 2024).

Conversely, deletion of all CTCF sites at a TAD boundary between the murine *Sox9* and *Kcnj2* loci results in some loss of insulation between the two TADs, which was further exacerbated by deletion of additional intra-TAD CTCF sites, eventually resulting in TAD fusion (Despang et al. 2019). However, this did not result in a detectable change in the pattern of *Sox9* expression or an overt phenotype in mice. Similarly, deletion of individual CTCF sites at the *Shh* locus TAD boundaries did not result in dysregulated *Shh* expression or developmental phenotypes in mice (Williamson et al. 2019). In contrast, a 12 kb deletion within the *Shh* TAD, encompassing three CTCF sites, results in a limb truncation phenotype in humans but not in mice (Ushiki et al. 2021), and deletion of intra-TAD CTCF sites reduces *Shh* expression in the mouse limb (Paliou et al. 2019).

There is some evidence that the ability to activate across topological boundaries may be enhancer-specific. Some enhancers are still able to activate *Sox2* in differentiation and development across topological boundaries created by the insertion of arrays of CTCF sites, whereas the ability of other enhancers to activate the same gene during development is impeded by these same CTCF insertions (Chakraborty et al. 2023).

*Shh* expression in the zone of polarizing activity (ZPA) of the distal posterior mesenchyme of the developing mouse limb is driven by the ZRS enhancer, which is located within intron 5 of the ubiquitously expressed *Lmbr1* at the opposite end of the 900 kb TAD from *Shh* itself (Fig. 1A; Lettice et al. 2003; Sagai et al. 2005). However, we have previously identified that ZRS also appears to be able to drive low-level expression of *Mnx1*, located 150 kb away in the adjacent TAD, in the ZPA of the developing limb bud (Williamson et al. 2019). *Mnx1* is primarily expressed in developing motor neurons of the neural tube, driven by proximal enhancers, and Mnx1 function is required to specify motor neuron identity (Arber et al. 1999; Wichterle et al. 2002; Nakano et al. 2005). Consistent with in situ hybridization data (Rock et al. 2007; Williamson et al. 2019), a transgene inserted at the *Mnx1* locus gives detectable lacZ staining in the ZPA of the E11.5 limb (Arber et al. 1999), and a lacZ reporter inserted 24 kb upstream of the *Lmbr1* promoter, just beyond the *Shh* TAD boundary, shows evidence of weak expression driven by ZRS in the limb and by *Mnx1* enhancers in the neural tube (Anderson et al. 2014). There is no reported defect in limb development in *Mnx1* knockout mice, suggesting that low-level *Mnx1* expression in limb development is bystander activation, likely driven by ZRS. This is all the more remarkable because this activation is in animals across an intact TAD boundary.

**Figure 1.**
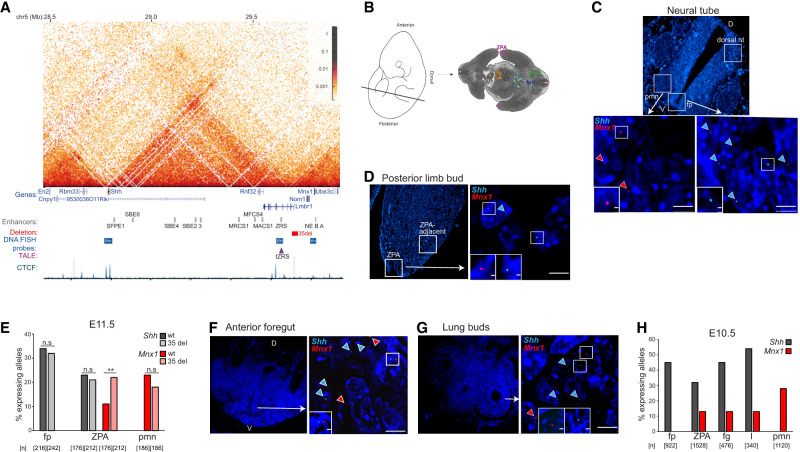
Long-range *Shh* enhancers can activate transcription at *Mnx1* in Shh-expressing tissues. (*A*) Hi-C heat map, created using HiGlass, of the *Shh* TAD from wild-type mESCs, at 16 kb resolution. Data are from Boyle et al. (2020). Genes, *Shh* and *Mnx1* enhancers, 35 kb deletion, binding locations of DNA-FISH fosmid probes, position of the TALE target, and the CTCF ChIP-seq track, are shown *below* the heat map. Genome coordinates (in megabases): mm9 assembly of the mouse genome. (*B*) Mouse embryo cartoon (*left*) indicating the position and orientation of the tissue sections analyzed (*right*). Highlighted are the zone of polarizing activity (ZPA; purple), floorplate (fp; blue), pre-motor neurons (pmn; green), foregut (fg; orange), lung buds (l; light blue), and nonexpressing limb and neural tube tissue (red boxes). (*C*) Representative images of tissues (*top*) and nuclei (*bottom*) showing RNA-FISH signal at *Shh* (cyan) and *Mnx1* (red) in the floorplate (fp) and pre-motor neurons (pmn) of the neural tube. (D) Dorsal, (V) ventral. Scale bars, 5 µm. (*D*) As in *C* but in the ZPA of the posterior forelimb bud. (*E*) The percentage of alleles with *Shh* and *Mnx1* RNA-FISH signal in wild-type (wt) and 35 kb deletion (35 del) E11.5 mouse embryos in expressing tissues of the neural tube (floorplate [fp] for *Shh* and pre-motor neurons [pmn] for *Mnx1*) and the posterior limb bud (ZPA for both genes). The data were compared using a two-sided Fisher's exact test. (n.s.) Not significant, (**) *P* < 0.01. The number of alleles scored (*n*) is shown *below*. Data for a biological replicate are shown in Supplemental Figure S1A. Proportions transcribed and statistical data are shown in Supplemental Table S1. (*F*,*G*) Representative images of tissues and nuclei showing RNA-FISH signal for *Shh* (cyan) and *Mnx1* (red) in the ventral anterior foregut (*F*) and lung bud (*G*). Scale bars, 5 µm. (*H*) The percentage of *Shh*-expressing alleles in the floorplate (fp) and *Mnx1*-expressing alleles in the pmns in comparison with expression of both genes in the ZPA, foregut (fg), and lung buds (l) of an E10.5 embryo assayed by RNA-FISH. Data for a biological replicate are shown in Supplemental Figure S1B. The number of alleles scored (*n*) is shown *below*.

Here we investigated the coactivation of *Shh* and *Mnx1* by enhancers within the Shh TAD using nascent RNA-FISH to determine whether transcription at *Shh* and *Mnx1* alleles on the same chromosome can co-occur or is mutually exclusive and DNA-FISH to examine the spatial context in which such activation occurs. We explored the role of cohesin in activation at *Shh* and *Mnx1* by synthetically activating enhancers in the *Shh* TAD. Our results have implications for models of enhancer–promoter communication and the role of spatial proximity, loop extrusion, and TAD boundaries in developmental gene regulation.

## Results

### *Mnx1* transcription is detected in some *Shh*-expressing tissues

*Shh* and *Mnx1* have very different zones of expression in the developing murine neural tube. *Shh* is expressed in the floorplate, regulated by enhancers located close to, and within, the *Shh* gene at the centromeric end of the TAD (Anderson et al. 2014). *Mnx1* in the adjacent TAD is expressed in the developing motor neurons of the neural tube, driven by enhancers just upstream of the *Mnx1* promoter (Fig. 1A–C; Nakano et al. 2005). *Shh* expression in the ZPA, at the posterior distal margin of the developing limb bud, is driven by ZRS 900 kb upstream of *Shh* at the opposite end of the *Shh* TAD (Lettice et al. 2003; Sagai et al. 2005). ZRS is only 150 kb from *Mnx1* in the adjacent TAD (Fig. 1A). Evidence for *Mnx1* expression in the developing mouse limb bud comes from quantitative RT-PCR (qRT-PCR), in situ hybridization (Williamson et al. 2019), and lacZ staining (Arber et al. 1999). To confirm that this expression is in the mesenchymal cells of the ZPA underlying the surface ectoderm, we performed dual-color RNA-FISH to detect nascent *Shh* and *Mnx1* transcripts in E10.5 and E11.5 mouse embryos. Both *Shh* transcription and *Mnx1* transcription were detected in the ZPA (Fig. 1D), with nascent *Mnx1* RNA detected for 9%–20% of alleles, which is approximately half the frequency for *Shh* and significantly lower than the frequency of *Mnx1* transcription in pre-motor neurons (Fig. 1E,H; Supplemental Fig. S1A,B; Supplemental Table S1). Expression from both genes was minimal in the dorsal neural tube and locations adjacent to the ZPA in the posterior limb bud (Fig. 1C,D; Supplemental Fig. S1C). There was negligible detection of *Mnx1* transcription driven by the *Shh* SFPE1 enhancer in the floorplate or of *Shh* in pre-motor neurons of the neural tube driven by *Mnx1* enhancers NE, B, and A, indicating that the enhancers proximal to each gene are unable to activate the other gene (Supplemental Fig. S1C; Supplemental Table S2). These data would be consistent with ZRS being able to activate *Mnx1* in the ZPA despite the presence of an intervening TAD boundary.

We have previously shown that a 35 kb deletion (35 del) of the TAD boundary separating ZRS from *Mnx1*, including the first two exons of *Lmbr1* and extending 13 kb upstream of the *Lmbr1* promoter, leads to increased *Mnx1* expression in the ZPA as detected by in situ hybridization to embryonic limb sections and qRT-PCR on dissected limb buds (Williamson et al. 2019). We confirmed a significant increase in transcription frequency at *Mnx1* in the ZPA of 35 del homozygous embryos by RNA-FISH, with no apparent effects on *Shh* expression (Fig. 1E; Supplemental Fig. S1A; Supplemental Table S1).

A bit further into the Shh TAD from ZRS, the MACS1 enhancer, located in intron 8 of *Rnf32* (Fig. 1A), drives *Shh* expression in the epithelial linings of the laryngotracheal tube, stomach, and lungs (Sagai et al. 2017a). We therefore considered that *Mnx1* might also be responsive to this *Shh* enhancer. Indeed, dual-color RNA-FISH revealed transcription at *Mnx1*, along with the expected *Shh* expression, in the epithelium of the ventral foregut and the lung bud of E10.5 embryos (Fig. 1F,G). Although the proportion of active *Mnx1* alleles is approximately one-third of those of *Shh* in these tissues (Fig. 1H; Supplemental Fig. S1B), the frequency of transcription at *Mnx1* in these epithelial cells, as well as in the ZPA, is significantly greater than in nonexpressing *Shh* tissue and in the floorplate, where *Shh* expression is driven by its proximal enhancers >1 Mb away from *Mnx1* (Supplemental Table S2). These data suggest that transcription from *Mnx1* can be activated by *Shh* enhancers located at least a few hundreds of kilobases away in the neighboring TAD.

This prompted us to explore the extent to which enhancers even further into the *Shh* TAD might be able to activate transcription at *Mnx1*. We focused on a further five *Shh*-expressing tissues located within the oral cavity and brain (Supplemental Fig. S1D,E). The next two enhancers beyond MACS1—MFCS4 and MRCS1 (Fig. 1A)—are active in the pharynx epithelium and soft palate epiglottis, respectively (Sagai et al. 2017b). Transcription at *Mnx1* can also be detected in these tissues and is increased in 35 del embryos, with no effect on *Shh* expression (Supplemental Fig. S1F; Supplemental Tables S1, S2). Remarkably, we could also detect transcription at *Mnx1* within the telencephalon and diencephalon forebrain regions, where *Shh* is regulated by the SBE3 and SBE2 enhancers, respectively, located in the middle of the *Shh* TAD (∼600 kb away from *Mnx1*) (Supplemental Fig. S1F; Supplemental Tables S1, S2; Jeong et al. 2006). The frequency of transcription detected at *Mnx1* alleles in the telencephalon (14%–15%) was similar to that in the oral cavity, foregut, lung buds, and limb. TAD boundary deletion (35 del) also led to increased transcription at *Mnx1* in the telencephalon. The frequency of transcription at *Mnx1* alleles was low in the diencephalon (6%–8%) but still significantly higher than in the nonexpressing control tissue. This was not the case when we assayed transcription at *Mnx1* in the floorplate of the neural tube at the base of the hindbrain. *Shh* expression here is under the control of the SBE6 enhancer located 900 kb from *Mnx1* (Fig. 1A; Benabdallah et al. 2016). The low frequency of transcribing *Mnx1* alleles detected by RNA-FISH in the neural tube was not significantly different from that in the control dorsal part of the neural tube at the base of the hindbrain (Supplemental Fig. S1F; Supplemental Tables S1, S2). We conclude that transcription at *Mnx1* can be activated from enhancers located at the center of a neighboring TAD but not from enhancers further away at the distal end of that TAD.

### *Mnx1* and *Shh* can be transcribed concurrently from the same allele

Contrary to enhancer–promoter looping models that predict noncoincident transcriptional bursts from genes sharing the same enhancer, studies in *Drosophila* show coordinated bursting from two reporter genes under the control of a single enhancer (Fukaya et al. 2016; Lim and Levine 2021). Whether there could be concurrent transcription from two endogenous genes driven by the same enhancer has not been explored.

**Figure 2.**
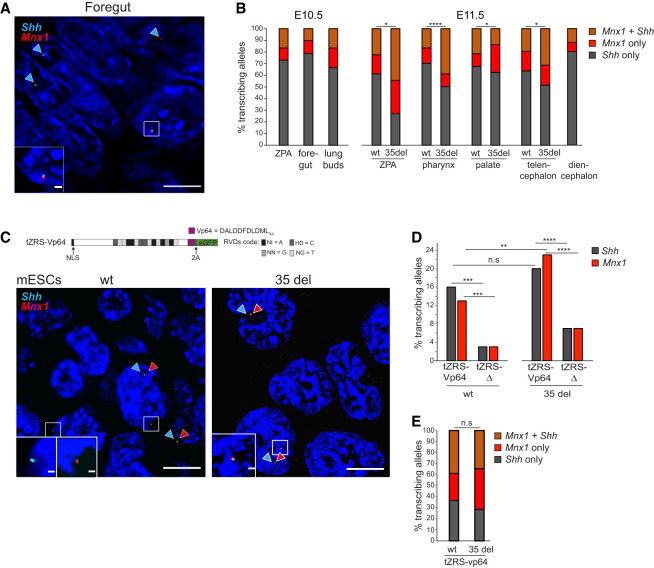
Adjacent RNA-FISH signals signify concurrent transcription at *Shh* and *Mnx1* driven by long-range enhancers. (*A*) Representative image of foregut nuclei from E10.5 embryos showing RNA-FISH signal at *Shh* (cyan) and *Mnx1* (red). Scale bars, 5 µm. (*B*) Bar graph showing the percentage of active alleles transcribing at both *Shh* and *Mnx1* (brown) or at *Shh* (gray) or *Mnx1* (red) alone in the ZPA, ventral foregut, and lung bud epithelial cells of a wild-type (wt) E10.5 embryo (*left*) and in the ZPA, pharynx, palate, telencephalon, and diencephalon of wt and 35 del E11.5 embryos (*right*). Coactivation at both genes versus activation of a single gene at expressing alleles of the wt and 35 del E11.5 embryos was compared using a two-sided Fisher's exact test. (*) *P* ≤ 0.05 and *P* > 0.01, (****) *P* < 0.0001. Data from a biological replicate are shown in Supplemental Figure S2A. Coactivation proportions in E11.5 wild-type and 35 del cells and statistical data are shown in Supplemental Table S3. The proportion of transcribed alleles where either gene alone or both together were detected and statistical analysis on the significance of coactivation by a single enhancer for E10.5 and E11.5 tissues are shown in Supplemental Table S4. (*C*, *top*) Schematic of the TALE-Vp64 construct used to target the ZRS (tZRS-Vp64) enhancer. (NLS) Nuclear localization sequence, (2A) self-cleaving 2A peptide. Repeat variable diresidue (RVD) code is displayed at the *right* using the one-letter amino acid abbreviations. Equivalent TALE-Δ constructs lack the VP64 module. (*Bottom*) Representative images of mESC nuclei showing RNA-FISH signal for *Shh* (cyan) only, *Mnx1* (red) only, and both at the same allele. Scale bars, 5 µm. (*D*) The percentage of *Shh* transcribing and *Mnx1* transcribing alleles in wt (*left*) and 35 del (*right*) mESCs activated from ZRS targeted by either tZRS-Vp64 or tZRS-Δ. The data were compared using a two-sided Fisher's exact test. (n.s.) Not significant; (**) *P* < 0.01, (***) *P* < 0.001, (****) *P* < 0.0001. Data from a biological replicate are shown in Supplemental Figure S2B. The number of alleles scored, proportions transcribed, and statistical data are shown in Supplemental Table S5. (*E*) As in *B* but for wt and 35 del mESCs transfected with tZRS-Vp64. Data from a biological replicate are shown in Supplemental Figure S2C. Statistical data comparing wt and 35 del are shown in Supplemental Table S3. Statistical analysis of the frequency of concurrent activation by tZRS-Vp64 of *Shh* and *Mnx1* on the same allele versus different alleles in wt and 35 del mESCs is shown in Supplemental Table S4.

We therefore investigated whether transcription at *Mnx1* and *Shh* originates from the same or alternate chromosome. In the ZPA, foregut, lung buds, oral cavity, and forebrain, at least half of *Mnx1* RNA-FISH signals are at alleles that also show closely apposed signal for the *Shh* nascent transcript, which we consider indicates transcription from the same (in *cis*) chromosome (Fig. 2A,B; Supplemental Fig. S2A). An even higher proportion of *Mnx1* transcribing alleles also transcribes *Shh* in del 35 embryonic tissues, though for the pharynx, palate, and telencephalon this only reached statistical significance for one biological replicate (Fig. 2B; Supplemental Fig. S2A; Supplemental Table S3).

These data suggest that the ZRS, MFCS4, and SBE3 enhancers may be able to concurrently activate transcription at two loci on the same chromosome. To test whether there was a tendency of concurrent transcription on the same chromosome, only nuclei with exactly one RNA-FISH signal at each locus are informative. For these nuclei, we scored for in how many of them there was transcription at both *Shh* and *Mnx1* in *cis* and for how many nuclei showed transcription at each locus but happening on different chromosomes (in *trans*). Replicates were merged due to the reduced number of cells suitable for this analysis. We tested statistically whether there was an excess of nuclei showing expression in *cis* using logistic regression, fitting generalized linear models with a binomial link function to assess both the level of significance (if any) and the direction of the association; i.e., whether alleles were preferentially expressed on the same or on different chromosomes (see the Materials and Methods). No tissue or cell type examined showed preferential transcription of *Shh* and *Mnx1* on different chromosomes, providing no evidence in support of an enhancer working exclusively on one gene at a time (Supplemental Table S4). In contrast, in most *Shh*-expressing tissues of E11.5 embryos and in E10.5 foregut tissue, this analysis supports preferential transcription at *Shh* and *Mnx1* alleles on the same chromosome at the same time (Supplemental Table S4).

ZRS and MACs1 are relatively large complex enhancers (>800 bp) that bind a cocktail of transcription factors in developing tissues (Lettice et al. 2012, 2017; Peluso et al. 2017; Sagai et al. 2017a; Rankin et al. 2021; Huang et al. 2023). However, we have shown that in mouse embryonic stem cells (mESCs), transcription at *Shh* can also be induced by the recruitment of single synthetic transcription factors to either the promoter of *Shh* or to its long-range enhancers, including ZRS (Benabdallah et al. 2019; Kane et al. 2022). We therefore investigated whether TAL effector (TALE)-directed recruitment of Vp64 to ZRS could also induce transcription at *Mnx1* in mESCs. We have previously shown that these TAL-Vp64 tools also induce histone acetylation (H3K27ac) locally at the site of TAL binding and at the target gene, consistent with p300 recruitment, but this does not spread more generally across the region (Benabdallah et al. 2019).

Compared with a control TAL lacking fusion to Vp64 (tZRS-Δ), TAL-Vp64 targeted to ZRS (tZRS-Vp64) could significantly induce transcription at both *Shh* and *Mnx1* in mESCs (Fig. 2C,D; Supplemental Fig. S2B). Although deletion at the TAD boundary near ZRS in 35 del mutant mESCs had no significant effect on the ability of tZRS-Vp64 to activate transcription from *Shh*, it significantly enhanced transcription at *Mnx1* (Fig. 2D; Supplemental Fig. S2B; Supplemental Table S5). Therefore, an enhancer (ZRS) activated by a single species of transcription factor (tZRS-Vp64) can activate transcription across a TAD boundary, and this is enhanced by a deletion encompassing that boundary. As in several *Shh*-expressing tissues of mouse embryos (Fig. 2B), analysis of RNA-FISH data indicated that tZRS-Vp64 can activate transcription concurrently at both *Shh* and *Mnx1* alleles on the same chromosome (Fig. 2E; Supplemental Fig. S2C; Supplemental Table S4).

### Concurrent transcription at *Mnx1* and *Shh* occurs in the context of a compact chromosome conformation

**Figure 3.**
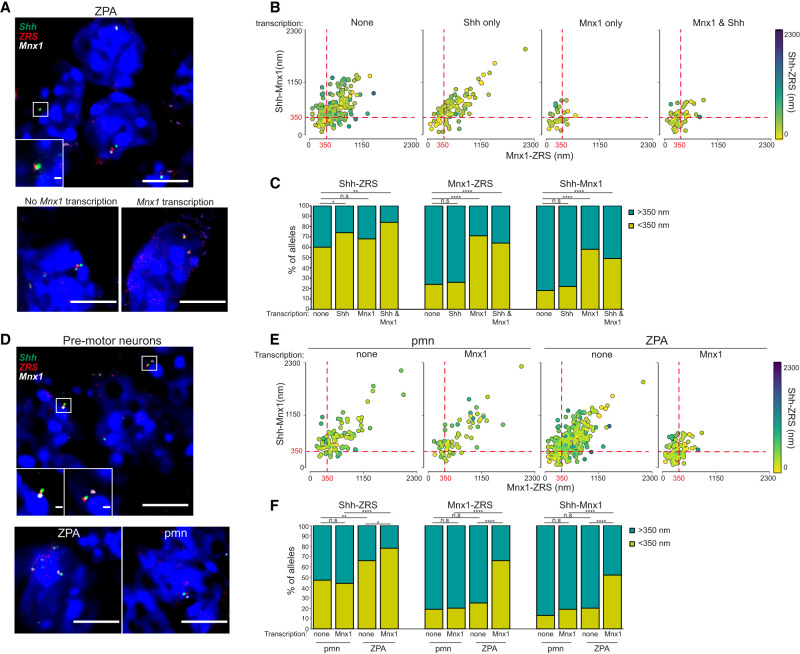
Spatial proximity of *Mnx1* to ZRS and *Shh* is optimal for limb bud ZPA transcription. (*A*, *top*) Representative image of ZPA nuclei from E10.5 embryos showing DNA-FISH signal for *Shh* (green), ZRS (red), and *Mnx1* (white). (*Bottom*) Representative nuclei showing *Mnx1* nontranscribing (*left*) and transcribing (*right*) alleles. Scale bars, 5 µm. (*B*) Scatter plots showing interprobe distances between each of the two probe pairs indicated on the *X-* and *Y*-axes, with the separation between the third pair indicated by the color (in the color bar) in ZPA cells at nontranscribing, *Shh* transcribing, *Mnx1* transcribing, and *Mnx1* and *Shh* transcribing alleles. Dashed red lines indicate alleles where *Shh*–*Mnx1* or *Mnx1*–ZRS interprobe distances are <350 nm. (*C*) Bar plots providing categorical analysis of the spatial relationship of *Shh*, ZRS, and *Mnx1* in ZPA cells at nontranscribing, *Shh* transcribing, *Mnx1* transcribing, and *Mnx1* and *Shh* transcribing alleles. Categories are as follows: <350 nm apart, the upper (75%) quartile distance of all *Shh* transcribing alleles (Supplemental Figure S3B), and >350 nm. Differences between nonexpressing and expressing alleles were identified using Fisher's exact test. (n.s.) Not significant, (*) *P* ≤ 0.05 and *P* > 0.01, (**) *P* ≤ 0.01, (****) *P* ≤ 0.0001. Data from two biological replicates are combined in *B* and *C*. Median and interquartile data for each of the two biological replicates are shown in Supplemental Figure S3C. Statistical analysis of the significance of spatial proximity at transcribing versus nontranscribing alleles and the proportion of interprobe distances <350 nm are shown in Supplemental Tables S6 and S7. (*D*, *top*) Representative image of neural tube pre-motor neuron nuclei from E10.5 embryos showing DNA-FISH signal for *Shh* (green), ZRS (red), and *Mnx1* (white). (*Bottom*) Images of a ZPA nucleus (*left*) and a pmn nucleus (*right*). Scale bars, 5 µm. (*E*) As in *B* but for pmns and ZPA cells at non-*Mnx1* transcribing and all *Mnx1* transcribing alleles. (*F*) As in *C* but in pmns and ZPA cells at non-*Mnx1* transcribing and *Mnx1* transcribing alleles. Differences between nontranscribing and transcribing alleles were identified using Fisher's exact test. (n.s.) Not significant, (*) *P* ≤ 0.05 and *P* > 0.01, (**) *P* ≤ 0.01, (****) *P* ≤ 0.0001. All data are from both biological replicates combined. Median and interquartile data for each of the two biological replicates are shown in Supplemental Figure S3D. Statistical analysis of the significance of spatial proximity at pmn *Mnx1* transcribing alleles versus pmn nontranscribing alleles and *Mnx1* transcribing and nontranscribing alleles in ZPA cells and the proportion of interprobe distances <350 nm are shown in Supplemental Tables S6 and S7.

To determine whether ZRS's ability to activate *Mnx1* in the neighboring TAD is linked to 3D genome organization, we used DNA-FISH for *Shh*, *ZRS*, and *Mnx1* on the limb bud tissue sections that we had analyzed previously by RNA-FISH (Fig. 3A; Supplemental Fig. S3A). This enabled us to compare spatial distances between *Shh* and *Mnx1* and the ZRS enhancer in the ZPA for alleles with no nascent transcription detected at either gene or those with transcription detected at one gene or the other or at both. Strikingly, transcription from *Mnx1* occurred in the context where the *Shh*–*Mnx1, Mnx1*–ZRS, and *Shh*–ZRS distances were all small. The *Mnx1*–*Shh* and *Mnx*–ZRS distances were larger at alleles where no transcription from *Mnx1* was detected (Fig. 3B; Supplemental Fig. S3B; Supplemental Table S6). Taking 350 nm as a cutoff, encompassing the upper quartile of interprobe distances between *Shh* and ZRS at all ZPA *Shh* transcribing alleles (Supplemental Fig. S3C), >50% of alleles in the limb bud show *Shh* and ZRS in proximity even when no *Shh* transcription is detected. This proportion increases further at *Shh* transcribing alleles (Fig. 3C). In contrast, <25% of alleles where no transcription at *Mnx1* is detected have *Mnx*–ZRS or *Mnx1*–*Shh* distances <350 nm, rising very significantly to 50%–70% at *Mnx1* transcribing alleles (Supplemental Table S7). This appears to be a consequence of ZRS-driven activation and not *Mnx1* transcription per se, as in the nuclei of pre-motor neurons, where *Mnx1* expression is driven from its own proximal enhancers (Fig. 1A), *Mnx*–ZRS and *Mnx1*-*Shh* distances are not different between *Mnx1*-expressing and nonexpressing alleles (Fig. 3D–F; Supplemental Fig. S3D; Supplemental Table S7). These data suggest that spatial proximity is important in the proposed ability of ZRS to activate transcription at *Mnx1*.

### Cohesin facilitates activation of *Mnx1* across the *Shh* TAD boundary

**Figure 4.**
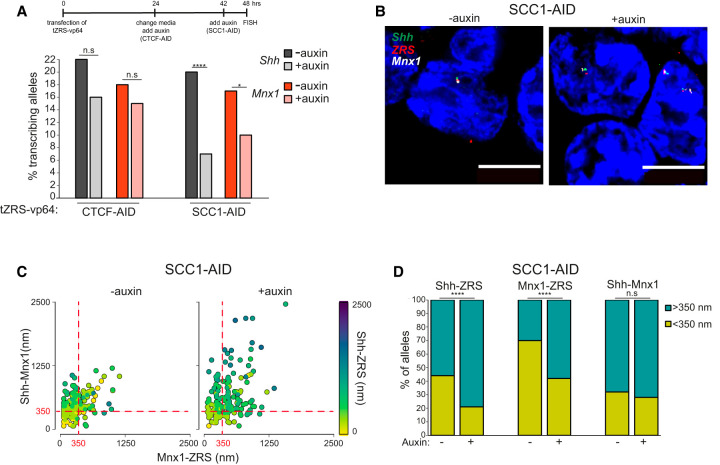
Cohesin optimizes *Mnx1* activation across the *Shh* TAD boundary. (*A*, *top*) Time course of TALE transfection and auxin treatment. (*Botom*) The percentage of *Shh* transcribing and *Mnx1* transcribing alleles in TALE transfected CTCF-AID cells (*left*) and SCC1-AID cells (*right*) either untreated (−auxin) or treated with 24 h (CTCF-AID) or 6 h (SCC1-AID) of auxin (+auxin) mESCs activated from the ZRS enhancer targeted by tZRS-Vp64. Data were compared using a two-sided Fisher's exact test. (n.s.) Not significant, (*) *P* ≤ 0.05 and *P* > 0.01, (****) *P* < 0.0001. Data from a biological replicate are shown in Supplemental Figure S4A. Values for the number of alleles scored and statistical evaluation are summarized in Supplemental Table S8. (*B*) Representative images of untreated (−auxin; *left*) and treated (+auxin; *right*) SCC1-AID nuclei showing DNA-FISH probe signal for *Shh* (green), ZRS (red), and *Mnx1* (white). Scale bars, 5 µm. (*C*) Scatter plots showing interprobe distances between each of the two probe pairs indicated on the *X*- and *Y*-axes, with the separation between the third pair indicated by the color (in the color bar) in untreated (−auxin; *left*) and treated (+auxin; *right*) SCC1-AID cells. Dashed red lines indicate alleles where *Shh*–*Mnx1* or *Mnx1*–ZRS interprobe distances are <350 nm. (*D*) Bar plots providing categorical analysis of the spatial relationship of *Shh*, ZRS, and *Mnx1* in SCC1-AID mESCs with (+) or without (−) auxin. Categories are as follows: <350 nm and >350 nm. Differences between cells with (+) or without (−) auxin were identified using Fisher's exact test. (n.s.) Not significant, (****) *P* ≤ 0.0001. Data from two biological replicates are combined in *C* and *D*. Median and interquartile data for each of the two biological replicates are shown in Supplemental Figure S4C. Statistical analysis of the significance of spatial proximity at transcribing versus nontranscribing alleles and the proportion of interprobe distances <350 nm are shown in Supplemental Tables S9 and S10.

It has been shown that cohesin and likely cohesin-mediated loop extrusion are required for enhancers to activate transcription at genes across large (several hundreds of kilobases) genomic distances (Calderon et al. 2022; Kane et al. 2022; Rinzema et al. 2022) but not for more short-range interactions. Indeed, we have shown previously that the ability of tZRS-Vp64 to activate transcription from *Shh* in mESCs depends on cohesin (SCC1/RAD21) but not on CTCF (Kane et al. 2022). We therefore set out to examine whether the ability of tZRS-Vp64 to activate transcription at *Mnx1* across the intervening TAD boundary was influenced by the degradation of CTCF or SCC1. We transfected tZRS-Vp64 into mESCs engineered to contain auxin-induced degron (AID)-tagged CTCF or SCC1 (Nora et al. 2017; Rhodes et al. 2020) and then degraded these tagged proteins by the addition of auxin as described previously (Kane et al. 2022). Published Hi-C and CTCF ChIP-seq data (Nora et al. 2017) show that both CTCF occupancy and the insulation score at the *Shh/Mnx1* TAD boundary are reduced upon auxin-induced degradation of CTCF (Kane et al. 2022). Consistent with deletion of CTCF sites in mice (Williamson et al. 2019) and our previous studies (Kane et al. 2022), CTCF degradation resulted in no significant change in the ability of tZRS-Vp64 to induce transcription at *Shh*, and this was also the case for transcription at *Mnx1* (Fig. 4A, Supplemental Fig. S4A; Supplemental Table S8). As shown previously (Kane et al. 2022), acute cohesin depletion very significantly blunted the ability of tZRS-Vp64 to activate transcription at *Shh*. It also reduced the ability of tZRS-Vp64 to induce transcription at *Mnx1* but to a lesser extent than for *Shh* (Fig. 4A; Supplemental Fig. S4A; Supplemental Table S8). In the absence of cohesin, tZRS-Vp64 more efficiently induced transcription at *Mnx1* than at *Shh*, likely a reflection of the smaller genomic distance separating ZRS and *Mnx1* compared with *Shh*.

We previously showed by DNA-FISH that significant decompaction of the *Shh* TAD occurs in the absence of cohesin (SCC1/RAD21) (Kane et al. 2022) and that spatial distances between ZRS and *Shh* increase. ZRS–*Mnx1* distances also significantly increase following cohesin depletion (Fig. 4B,C; Supplemental Fig. S4B; Supplemental Table S9), and the proportion of alleles with closely apposed ZRS–*Mnx1* (<350 nm) decreases (Fig. 4D; Supplemental Table S10). These data indicate that cohesin-mediated loop extrusion running through the intervening TAD boundary contributes to maintaining ZRS–*Mnx1* proximity.

Although there was an increased incidence of a few alleles with very large *Shh*–*Mnx1* distances upon Rad21/Scc1 degradation, the average distances and the proportion of alleles with closely apposed *Shh*–*Mnx1* loci (<350 nm) were not significant despite the large genomic distance (∼1 Mb) separating these two genes (Fig. 4C,D; Supplemental Fig. S4B,C; Supplemental Tables S9, S10). This is likely due to both genes being polycomb targets in mESCs and forming strong polycomb-mediated loops (Boyle et al. 2020; Williamson et al. 2023).

## Discussion

How the hundreds of thousands of enhancers in the mammalian genome activate their target genes, sometimes over intervening genes and large genomic distances, without inadvertently activating other nearby genes remains an unanswered question. Alignment of the large regulatory domains of developmental genes within TADs implicates 3D genome organization and cohesin-mediated loop extrusion in both facilitating and restraining enhancer action (Symmons et al. 2014, 2016; Andrey and Mundlos 2017). In particular, it has been proposed that intact TAD boundaries help to prevent enhancers in one TAD from activating genes in neighboring TADs, with genetic and epigenetic disruption of TAD boundaries giving rise to ectopic gene activation and disease phenotypes (Lupiáñez et al. 2015, 2016; Tsujimura et al. 2015; Flavahan et al. 2016, 2019; Hnisz et al. 2016).

However, experiments to test this hypothesis have not reached a consensus. In many cases, small deletions of TAD boundaries do not seem to affect gene regulation or result in mutant phenotypes (Despang et al. 2019; Paliou et al. 2019; Williamson et al. 2019). The insertion of ectopic CTCF sites can influence some enhancers but not others (Chakraborty et al. 2023). On the other hand, removal of CTCF sites at TAD boundaries can result in ectopic gene activation in cancer cell lines (Kim et al. 2024) and has been associated with Mendelian disease (Baudic et al. 2024). The phenotypic consequence of deleting regions containing CTCF sites close to long-range enhancers can also differ between mice and humans (Ushiki et al. 2021).

The *Shh* locus is an exemplar regulatory domain with >15 enhancers distributed across an ∼900 kb TAD of the human or mouse genomes, including in the introns of other genes. Aberrant *Shh* expression or developmental phenotypes are not seen in mice with deletion of individual CTCF sites at the *Shh* TAD boundaries (Paliou et al. 2019; Williamson et al. 2019). For example, deletion of CTCF sites at the TAD boundary closest to *Shh* (Fig. 1A) does not result in detectable ectopic *Shh* expression driven by the *En2* and *Cnpy1* enhancers located in the adjacent TAD (Song and Joyner 2000; Hirate and Okamoto 2006; Williamson et al. 2019). However, we previously noted that the other end of the *Shh* TAD (close to the ZRS *Shh* limb enhancer) mRNA for *Mnx1* (a gene located in the neighboring TAD) can be detected by in situ hybridization in the *Shh*-expressing ZPA of developing limb buds (Williamson et al. 2019).

Here, we used RNA-FISH, which detects nascent transcription, to confirm transcription at *Shh* or *Mnx1* in nuclei of the same cells of several Shh-expressing embryonic tissues, including the ZPA. These data suggest that several *Shh* enhancers located up to 500 kb from the ZRS-proximal Shh TAD boundary can act across that boundary to drive transcription at *Mnx1*. This is not the case for the SBE6 Shh enhancer located at the far end of the TAD ∼900 kb away from *Mnx1*. Consistent with our observations, a lacZ reporter transgene (SBLac936) inserted just beyond the *Shh* TAD boundary shows evidence of weak expression driven by ZRS in the limb and by *Mnx1* enhancers in the neural tube (Anderson et al. 2014).

In *Shh*-expressing tissues (the ZPA, pharynx, palate, and telencephalon), transcription at *Mnx1* is further enhanced by a 35 kb deletion that removes the TAD boundary separating ZRS from *Mnx1*. This suggests that the TAD boundary near the *Lmbr1* promoter does have some ability to insulate *Mnx1* from the influence of the *Shh* enhancers located within a few hundred kilobases. The deletion also reduces the genomic distance between the ZRS and Mnx1 from 150 to 115 kb, which could plausibly enhance the ability of the ZRS to activate *Mnx1*. However, this mechanism is less likely to explain increased activation at *Mnx1* by more distant enhancers such as SBE3, which is located ∼600 kb away.

Nascent transcription at both *Mnx1* and *Shh* could be detected concurrently on the same chromosome, often at a higher frequency than expected by chance. These findings suggest that several *Shh* enhancers, including the ZRS, can concurrently activate transcription at two genes across an intact but porous TAD boundary. This is consistent with the observation of simultaneous transcription of two reporter genes by a shared enhancer in *Drosophila* embryos (Fukaya et al. 2016) but, to our knowledge, is the first report of apparently concurrent transcriptional activation of two endogenous mammalian genes by the same enhancer across a TAD boundary.

It has been suggested that sequences within ZRS recruit specific transcription factors that endow the enhancer with the ability to act over large genomic distances in the developing limb (Lettice et al. 2014; Bower et al. 2024). However, we also detected nascent transcription from *Mnx1* in other tissues of the developing embryo where *Shh* expression is driven by several other enhancers, so the ability to activate across a TAD boundary is not limited to ZRS. We also demonstrated concurrent activation of *Mnx1* and *Shh* nascent transcripts using a synthetic transcription factor (tZRS-Vp64) targeted to ZRS in mESCs. This suggests that activation of transcription over long genomic distances and across a TAD boundary is not dependent on the recruitment of a specific cocktail of transcription factors.

Recently, it has been suggested that the stacking of TAD boundaries can bring regulatory elements in adjacent TADs into proximity, facilitating cross-boundary gene regulation (Hung et al. 2024). However, *Mnx1* is 100 kb from the ZRS TAD boundary, and we show that *Mnx1* can also be activated by Shh enhancers that are themselves several hundred kilobases from that TAD boundary. We therefore consider it unlikely that TAD boundary stacking is responsible for facilitating Shh enhancer–*Mnx1* communication.

**Figure 5.**
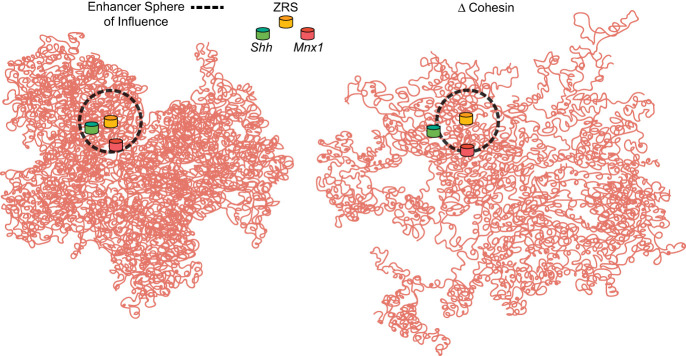
Cohesin and the ZRS sphere of influence. (*Left*) Cohesin-mediated chromatin compaction of the *Shh* TAD enables regulatory signals from the ZRS enhancer to reach and activate *Shh* >850 kb away and also enables some activation at *Mnx1*, which is much closer to ZRS genomically but located at the other side of a TAD boundary. (*Right*) Acute loss of cohesin results in a decompaction of the chromatin domain, putting *Shh* beyond the reach of signals from ZRS and considerably reducing the opportunity for *Mnx1* to be within the enhancer's “sphere of influence”. (Adapted from Gabriele et al. 2022. Adapted with permission from AAAS.)

We show that transcription activation of *Mnx1* from ZRS in the developing limb occurs in the context of a compact chromatin conformation, where *Mnx1* is close (<350 nm) to both *Shh* and ZRS. This is not the case when *Mnx1* transcription is being driven by its own proximal enhancer in pre-motor neurons. Together with the observation of concurrent transcription at *Mnx1* and *Shh*, which does not seem compatible with classical enhancer–promoter looping models (Lim and Levine 2021), this result is more consistent with a model in which enhancers nucleate the formation of transcription clusters or hubs that can activate transcription at genes coming into their sphere of influence (Fig. 5). We have previously shown that within the *Shh* TAD, cohesin but not CTCF is required for a synthetic transcription factor to activate *Shh* transcription from a large genomic distance (hundreds of kilobases) (Kane et al. 2022). In the absence of cohesin and presumably cohesin-mediated loop extrusion, decompaction of the TAD was observed, suggesting that one of the functions of loop extrusion is to compact large chromatin domains and thereby maintain target genes within the sphere of influence of enhancers located distant in the linear genome. Here we show that this also applies across a TAD boundary; the ability of a ZRS targeted transcription factor to activate transcription at *Mnx1* is attenuated when cohesin is degraded (Fig. 5). Interestingly, this is to a lesser extent than the influence of cohesin degradation on activation at *Shh*, such that transcription is now detected at a higher proportion of *Mnx1* alleles compared with *Shh*. This likely reflects the shorter genomic distance (150 kb) separating *Mnx1* from ZRS compared with *Shh* (850 kb).

Consistent with our previous genetic analysis of CTCF sites at the *Shh* locus in cells and in mice (Williamson et al. 2019), we detected no significant increase in ZRS-driven transcription at *Mnx1* when CTCF is degraded even though the latter results in reduced insulation at this TAD boundary, as assayed by Hi-C (Nora et al. 2017; Kane et al. 2022). In cancers, loss of CTCF binding due to altered DNA methylation has been suggested to result in ectopic enhancer-driven expression of cancer driver genes (Flavahan et al. 2016; 2019; Kim et al. 2024). It will therefore be important to better understand the different factors that contribute to functional TAD boundaries at different sites in the mammalian genome and in different cellular contexts.

Our findings suggest that not all TAD boundaries act as absolute barriers to enhancer function and that “ectopic” activation of genes from enhancers in adjacent TADs depends to some extent on the action of cohesin and presumably loop extrusion, resulting in spatial proximity of the enhancer and “ectopic” target gene. In the situation studied here (the apparent ectopic activation of *Mnx1* by enhancers in the *Shh* TAD), this appears to have no discernible biological function. The extent of such ectopic activation, or indeed other regulatory influences (Tsujimura et al. 2015; Galupa et al. 2020), across TAD boundaries in the mammalian genome remains to be explored, but it is interesting to speculate that it may provide a framework on which evolution can act to drive new patterns of gene expression.

## Materials and methods

### Mouse strains and embryo sectioning

Embryos were collected from timed matings of wild-type and the 35 del (Williamson et al. 2019) mouse lines and were fixed, embedded, sectioned, and processed for FISH as described previously (Morey et al. 2007; Williamson et al. 2019), except that sections were cut at 8 μm.

All mouse work was approved by the University of Edinburgh Animal Welfare and Ethics Review Board and was conducted under the authority of Home Office licenses.

### Cell culture and treatments

The mouse embryonic stem cells (mESCs) used were wild-type E14 (parental line of the CTCF-AID cells), CTCF-AID (Nora et al. 2017), SCC1-AID (Rhodes et al. 2020), and the homozygous 35 del line (Williamson et al. 2019).

Feeder-free mESCs were cultured and transfected as described previously (Kane et al. 2022). Briefly 1.5 × 10^6^ mESCs were transfected with 14.5 μg of TALE plasmid and 26 μL of Lipofectamine 3000 reagent (Invitrogen L3000015) and seeded onto 0.1% gelatin-coated 10 cm dishes containing SuperFrost Plus adhesion glass slides. Fresh media was added after 24 h. After 48 h of transfection, slides were washed, fixed in 4% paraformaldehyde (pFa), and permeabilized in 70% ethanol for a minimum of 24 h (up to 1 week) at 4°C. For auxin-inducible protein degradation, cells from half of each TALE transfection were treated with 500 µM auxin, and half were left untreated for an internal control. Auxin was added to the CTCF-AID 24 h after transfection and left for a further 24 h, whereas the SCC1-AID cells received auxin 42 h after transfection and were treated for a further 6 h.

### RNA-FISH

Custom Stellaris RNA-FISH probes were designed against *Shh* nascent mRNA (pool of 48 22-mers designed to NCBI37/mm9: chromosome 5: 28,787,847–28,793,741) and *Mnx1* nascent mRNA (pool of 46 22-mers designed to chromosome 5: 29,800,772–29,804,270) using the Stellaris RNA-FISH probe designer (Biosearch Technologies, Inc.), following the manufacturer's instructions (https://www.biosearchtech.com/stellarisprotocols) and as described previously (Williamson et al. 2019).

### 3D DNA-FISH

Following RNA-FISH, slides were reprobed by DNA-FISH. After the removal of coverslips, slides were briefly washed in PBS and then for 5 min in 2× SSC at 75°C (80°C for mESCs) followed by denaturation in 70% formamide/2× SSC for 20 min at 75°C (50 min at 80°C for mESCs) before a series of alcohol washes (70% [ice-cold], 90%, and 100%). We used 160–240 ng of biotin- and digoxigenin-labeled and red-dUTP-labeled (Alexa fluor 594-5-dUTP; Invitrogen) or Green496-dUTP-labeled (Enzo Life Sciences) fosmid probes (Supplemental Table S11) per slide, with 16–24 µg of mouse Cot1 DNA (Invitrogen) and 10 µg of salmon sperm DNA. EtOH was added and the probe was air-dried. Hybridization mix containing deionized formamide, 20× SSC, 50% dextran sulfate, and Tween 20 was added to the probes for ∼1 h at room temperature. Probes in hybridization mix were denatured for 5 min at 70°C and reannealed for 15 min at 37°C before being added to the slides and incubated overnight at 37°C. Following a series of washes in 2× SSC at 45°C and 0.1× SSC at 60°C, slides were blocked in blocking buffer (4× SSC, 5% Marvel) for 5 min. Slides were incubated with antibody in a humidified chamber for 30–60 min at 37°C in the following order with 4× SSC/0.1% Tween 20 washes in between: fluorescein anti-dig FAB fragments (1:20 dilution; Roche 11207741910), fluorescein antisheep (1:100; Vector Laboratories FI-6000)/streptavadin Cy5 (1:10; Amersham PA45001, lot 17037668), biotinylated anti-avidin (1:100; Vector Laboratories BA-0300, lot ZF-0415), and streptavidin Cy5 (1:10). Slides were treated with 1:1000 dilution of 50 µg/mL DAPI stock for 5 min before mounting in VectaShield.

### Image acquisition and deconvolution

Slides from RNA-FISH and DNA-FISH were imaged on an epifluorescence microscope as described previously (Boyle et al. 2020). Step size for *z*-stacks was set to 0.2 µm. Hardware control and image capture were performed using Nikon Nis-Elements software (Nikon), and images were deconvolved using a calculated PSF with the constrained iterative algorithm in Volocity (PerkinElmer). RNA-FISH signal quantification was carried out using the quantitation module of Volocity (PerkinElmer). Number of transcribing alleles was calculated by segmenting the hybridization signals and scoring each nucleus as containing zero, one, or two RNA signals. DNA-FISH measurements were made using the quantitation module of Volocity (PerkinElmer), and only alleles with single probe signals were analyzed to eliminate the possibility of measuring sister chromatids.

### Statistical analysis

DNA-FISH interprobe distance data sets were compared using the two-tailed Mann–Whitney *U*-test, a nonparametric test that compares two unpaired groups. Differences in DNA-FISH data sets comparing categorical distributions, differences in the proportion of *Shh* and *Mnx1* transcribing alleles in wild-type and 35 kb deletion embryos, and mESCs transfected with either tZRS-Vp64 or tZRS-Δ, as well as comparisons between the proportion of transcribing alleles in SCC1-AID mESCs ± auxin transfected with tZRS-Vp64, were measured using Fisher's exact test. These statistical analyses were performed using GraphPad Prism 9.4.1 (Mann–Whitney, *t*-test) or an online GraphPad 2 × 2 contingency table (https://www.graphpad.com/quickcalcs/contingency1) (Fisher's).

To test statistically whether there was an excess of nuclei showing concurrent transcription at *Shh* and *Mnx1* in *cis*, we used logistic regression, a form of generalized linear regression model. As input, we used a data frame with only one column, “hm,” of ones and zeroes, denoting expression in *cis* and in *trans*, respectively. We then called R's built-in glm function as myModel <- glm(hm∼1, data = myDataFrame) for each data set. For each fit, we tested whether the model intercept was significantly different from zero by using the *z*-scaled test statistic returned by these models and converting it to a *P*-value; i.e., we inspected the output of summary(myModel) for each fit.

## Supplemental Material

Supplement 1
