# Supplementary material for: Bystander activation across a TAD boundary supports a cohesin-dependent transcription cluster model for enhancer function
Source: Genes Dev. 2025 Sep 1;39(17-18):1012–24. doi: 10.1101/gad.352648.125 (PMC12404194; doi:10.1101/gad.352648.125)
Supplement: Supplement 1 [file Supplemental_Material.pdf]

Supplemental Material

Supplemental Figures 1-4

Supplemental Tables 1-11

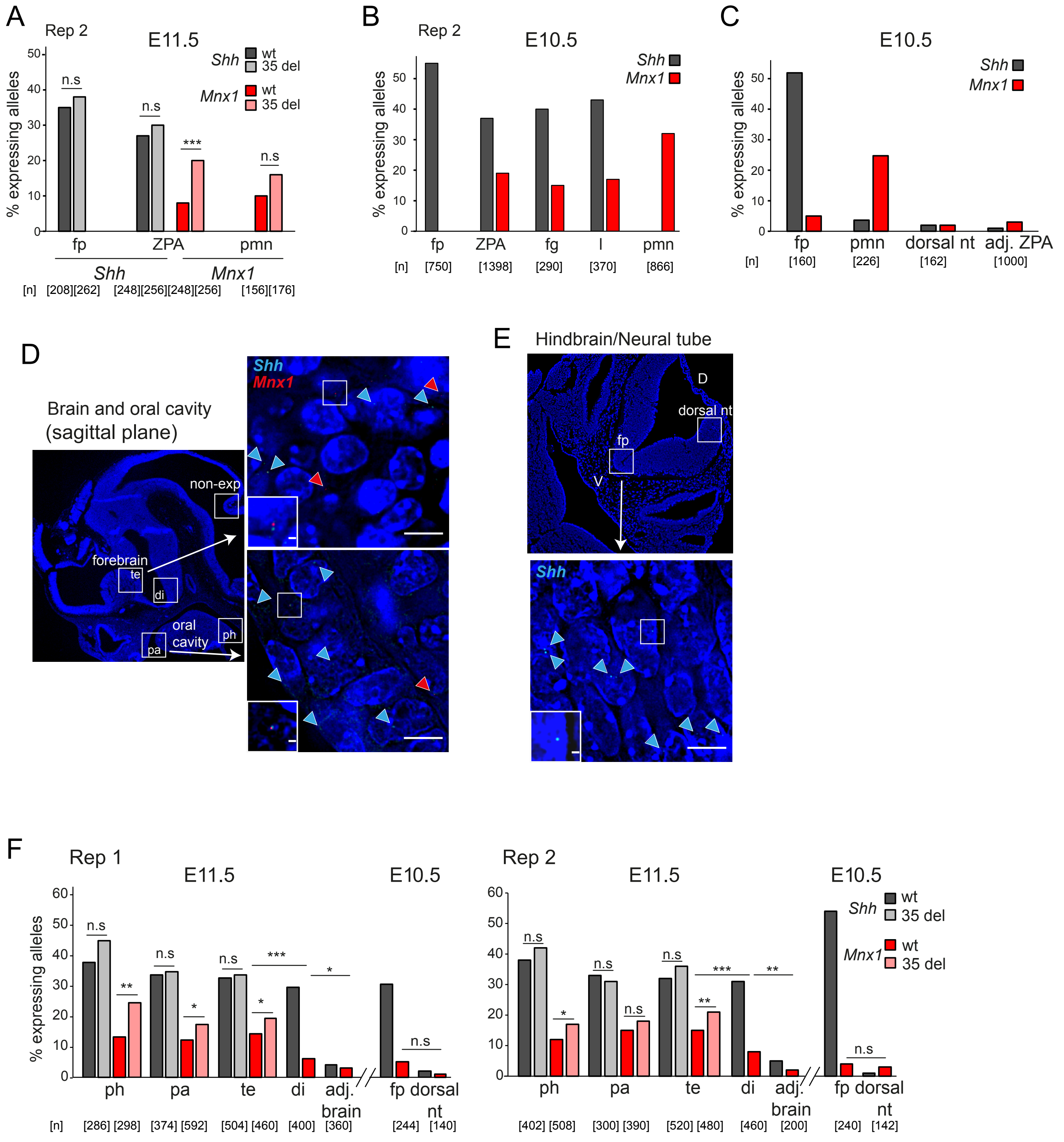

Supplemental Figure S1. Related to Figure 1. (A) Percentage of alleles with *Shh* and *Mnx1* RNA FISH signal in wild type (wt) and 35 kb deletion (35 del) E11.5 mouse embryos in tissues of the floorplate (fp), ZPA and pre-motor neurons (pmn). Number of alleles [n] scored are shown below. Data were compared using a two-sided Fisher's exact test; n.s., not significant; ***, $P < 0.001$. Data are for a biological replicate of Figure 1E. Proportions transcribed, and statistical data are in Supplemental Table S1. (B) % of *Shh*-expressing alleles in the fp and *Mnx1*-expressing alleles in the pmn in comparison to the expression of both genes in the ZPA, foregut (fg) and lung buds (l) of an E10.5 embryo. Data are for a biological replicate of Fig. 1H. Number of alleles scored [n] are shown below. (C) As in (B) for the fp, pmn, dorsal neural tube (nt) and locations adjacent (adj.) to the ZPA of wild type E10.5 mouse embryos. (D) Representative images of tissue (left) and nuclei (right) showing RNA-FISH signal at *Shh* (cyan) and *Mnx1* (red) in the telencephalon (te) and palate (pa) of the forebrain and oral cavit. D = dorsal, V = ventral, di = diencephalon, ph = pharynx, non-exp = non-expressing tissue. Scale bars, 5 μm. (E) As in (D) but for the floorplate (fp) of the hindbrain/neural tube, dorsal neural tube is non-expressing tissue. (F) As in (A) but for *Shh*-expressing and non-expressing tissues of the oral cavity and forebrain, and in the fp and dorsal neural tube at the base of the hindbrain in E10.5 embryos. Two biological replicates. The data were compared using a two-sided Fisher's exact test; n.s., not significant; *. $P \leq 0.05$ and > 0.01; **, $P < 0.01$; ***, $P < 0.001$. Data for two biological replicates. Proportions transcribed, and statistical data are in Supplemental Table S1.

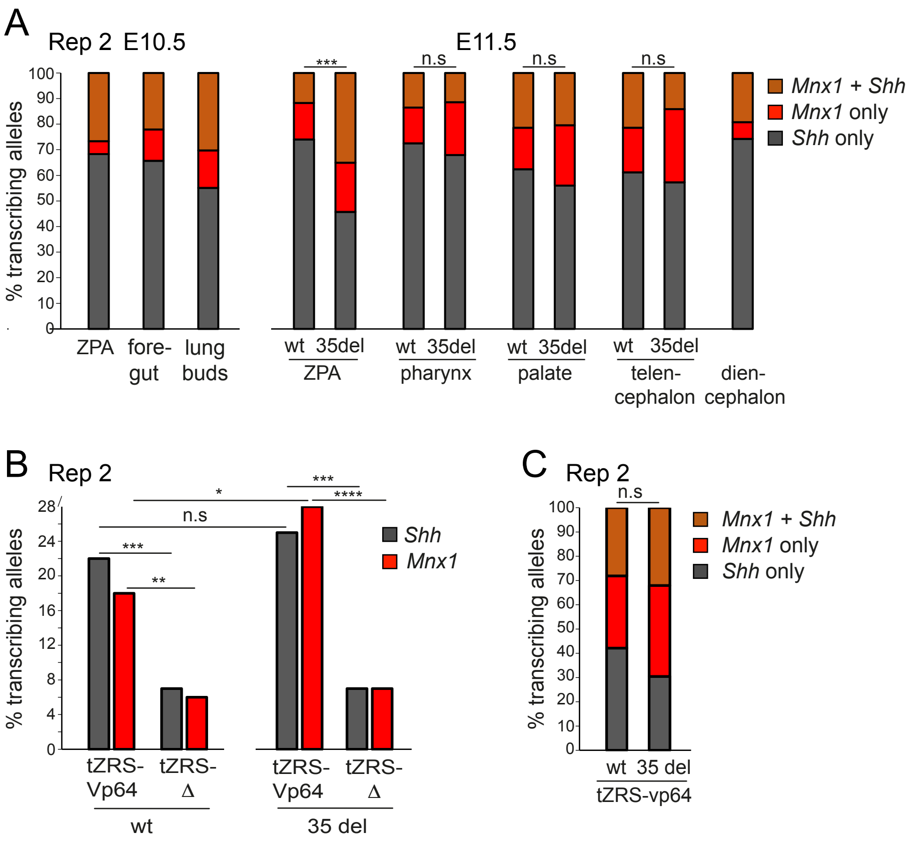

Supplemental Figure S2. Related to Figure 2. (A) Bar graph showing the proportion of active alleles transcribing at both *Shh* and *Mnx1* (brown), or at *Shh* (grey) or *Mnx1* (red) alone, in the ZPA, ventral foregut, and lung bud epithelial cells of a wild type E10.5 embryo (left) and in the ZPA, pharynx, palate, telencephalon and diencephalon of wild type (wt) and 35 kb deletion (35 del) E11.5 embryos (right). Co-activation at both genes versus activation of a single gene at expressing alleles of the wild type and 35 del E11.5 embryos was compared using a two-sided Fisher's exact test; ***, $P < 0.001$. Data are a biological replicate for Fig. 2B. Co-activation proportions in E11.5 wt and 35 del cells and statistical data are in Supplemental Table S3. Statistical analysis of the frequency of co-activation of Shh and Mnx1 on the same allele vs different alleles in E10.5 and E11.5 tissues are in Supplemental Table S4. (B) % of *Shh*- and *Mnx1*-transcribing alleles in wt (left) and 35 del (right) mESCs activated from the ZRS targeted by either tZRS-Vp64 or tZRS-Δ. The data were compared using a two-sided Fisher's exact test; n.s., not significant; *, $P \leq 0.05$ and > 0.01, **, $P < 0.01$; ***, $P < 0.001$; ****, $P < 0.0001$. Data are from a biological replicate for Fig. 2D. Number of alleles scored, proportions transcribed, and statistical data are in Supplemental Table S5. (C) As in (A) but in wt and 35 del mESCs transfected with tZRS-Vp64. Data are for a biological replicate of Fig. 2E. Co-activation proportions in wt and 35 deletion cells are in Supplemental Table S3. Statistical analysis on the significance of co-activation by the ZRS enhancer for E14 wt and 35 del mESCs are in Supplemental Table S4.

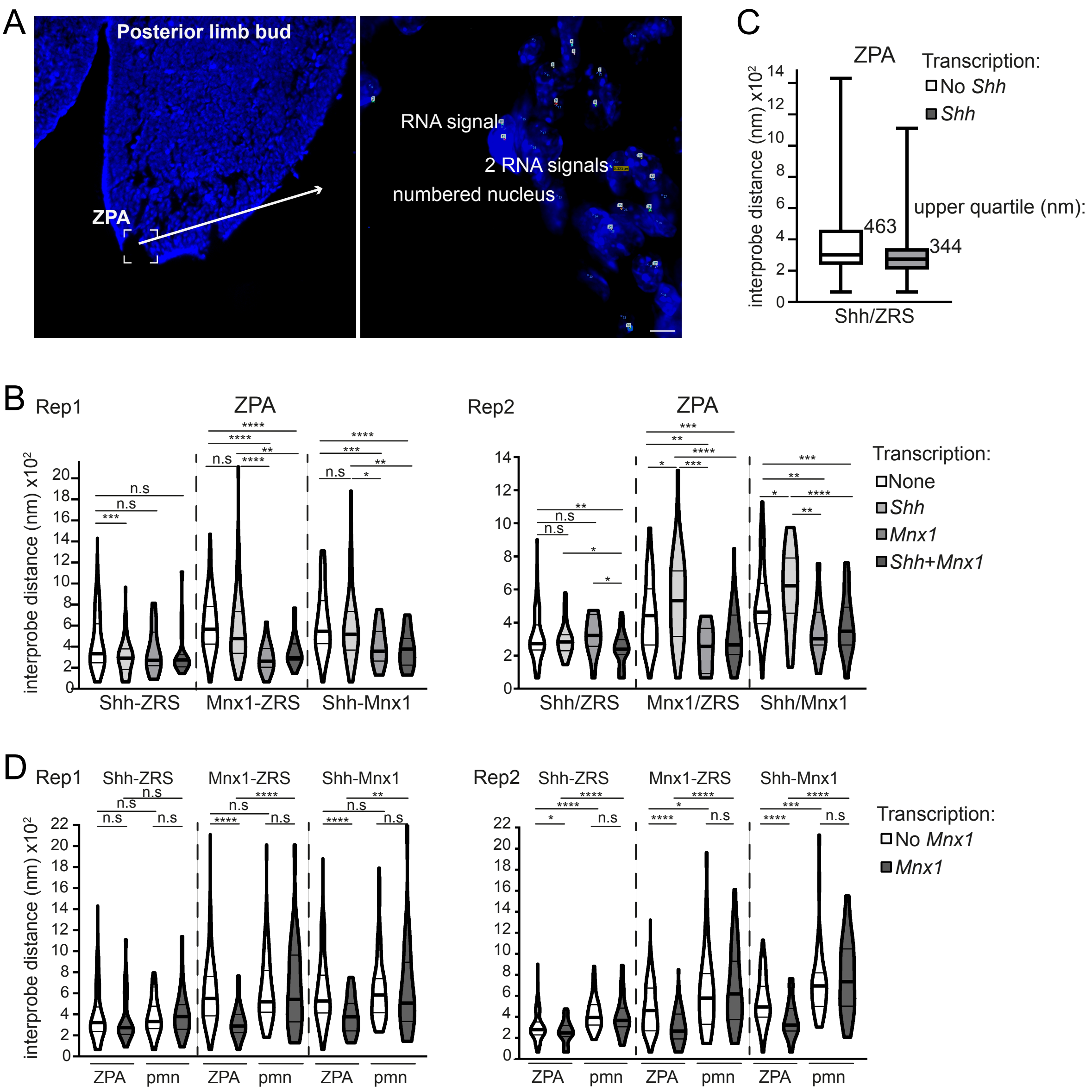

Supplemental Figure S3. Related to Figure 3. (A) Representative images of E10.5 limb bud tissue section (left) that had been analysed by RNA-FISH (right) prior to DNA-FISH. (B) Violin plots showing the distribution of interprobe distances (nm) between *Shh*-ZRS, *Mnx1*-ZRS, *Shh*-*Mnx1* probes in ZPA cells from two biological replicates at non-, *Shh*-, *Mnx1*- and *Mnx1* & *Shh*-transcribing alleles. (C) Box plots showing the distribution of *Shh*-ZRS interprobe distances (nm) at *Shh* non-transcribing and transcribing alleles in ZPA cells from the two biological replicates combined. The upper quartile distances for non-transcribing and transcribing tissues are shown, the latter value determining the <350 nm category for optimal enhancer spatial proximity for Fig. 3. (D) As in (B) but for pre-motor neuron (pmn) and ZPA cells at non-*Mnx1*- and all *Mnx1*-transcribing alleles. The data were compared using a two-sided Mann-Whitney U-test; n.s., not significant; *, $P \leq 0.05$ and > 0.01; **, $P < 0.01$; ***, $P < 0.001$; ****, p<0.0001. Values for number of alleles scored, median and inter-quartile distances, and statistical evaluation are summarised in Supplemental Table S6.

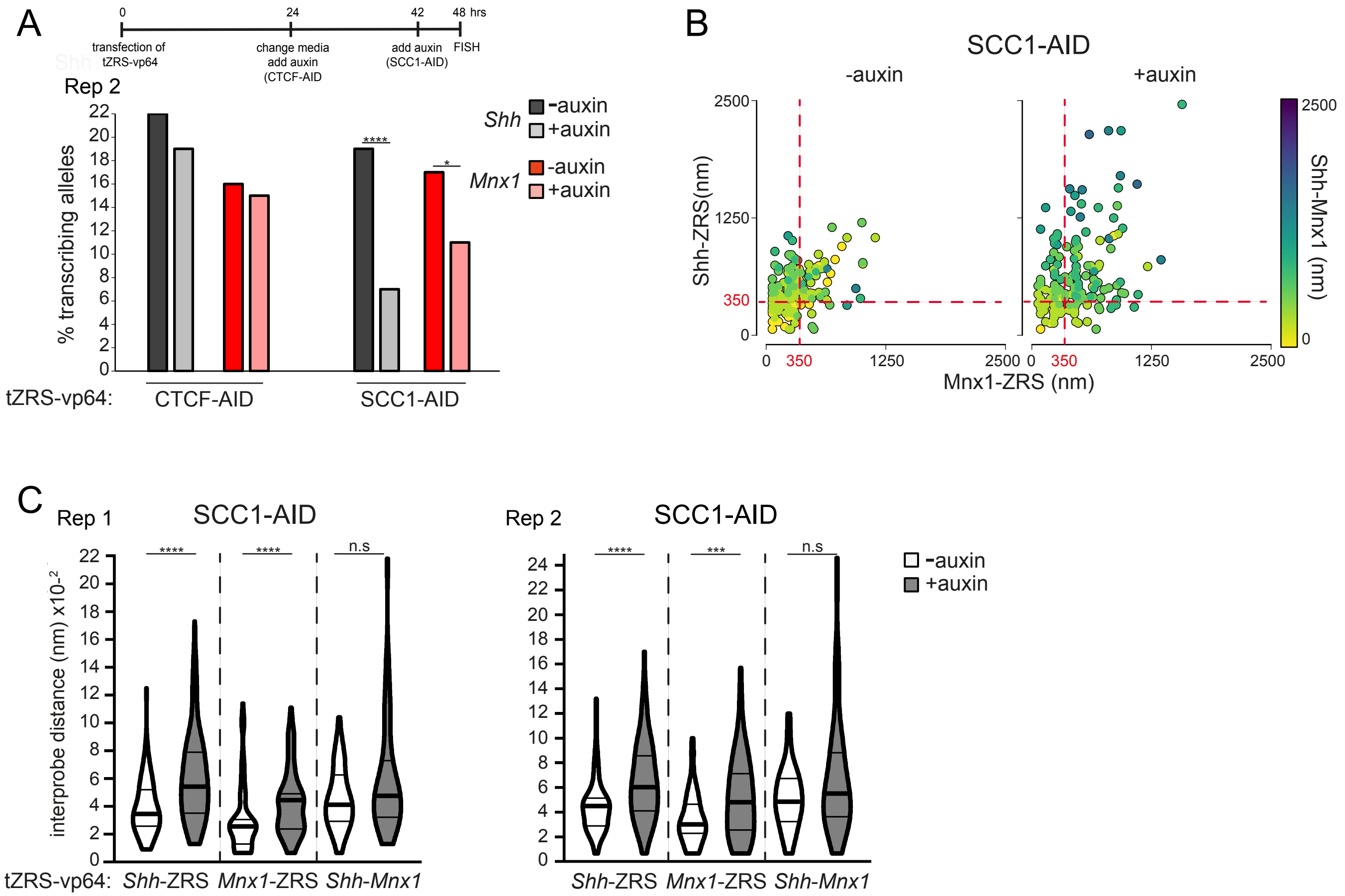

Supplemental Figure S4. Related to Figure 4. (A) Top; timecourse of TALE transfection and auxin treatment. Below; % of *Shh*- and *Mnx1*-transcribing alleles in TALE-transfected CTCF-AID cells (left) and SCC1-AID cells (right) either untreated (- auxin) or treated with 24 hours (CTCF-AID) or 6 hours (SCC1-AID) of auxin (+ auxin) in mESCs activated from the ZRS targeted by tZRS-Vp64. The data were compared using a two-sided Fisher's exact test; n.s., not significant; *, $P \leq 0.05$ and > 0.01; ****, $P < 0.0001$. Values for number of alleles scored and statistical evaluation are summarised in Supplemental Table S8. Data are from a biological replicate of Fig. 4A. (B) Scatter plots showing interprobe distances between each of the two probe pairs indicated on *x* and *y* axes with the separation between the third pair indicated by the colour (in the colour bar) in SCC1-AID cells - & + auxin. Dashed red lines indicates alleles where *Shh-Mnx1* or *Mnx1*-ZRS inter-probe distances are <350 nm. Same data as in Fig. 4C but with *Shh-Mnx1* rather than *Shh*-ZRS interprobe distances colour-coded. (C) Violin plots showing the distribution of interprobe distances (nm) between *Shh*-ZRS, *Mnx1*-ZRS, *Shh-Mnx1* probes in tZRS-Vp64-transfected SCC1-AID cells - or + auxin. Data from two biological replicates are shown. Data were compared using a two-sided Mann-Whitney U-test; n.s., not significant; ***, $P < 0.001$; ****, $P < 0.0001$. Values for number of alleles scored, median and inter-quartile distances, and statistical evaluation are summarised in Supplemental Table S9.

Table S1. Proportion of *Shh* and *Mnx1* transcribing alleles in wild type and 35kb deletion E11.5 limb bud ZPA, oral cavity, forebrain and neural tube tissues

Tissue	Genotype	Transcribing alleles (%) Number of alleles [ ]			
		Shh		***Mnx1***	
		Replicate 1	Replicate 2	Replicate 1	Replicate 2
**ZPA**	wt	23 [176]	27 [248]	11 [176]	8 [248]
	35 del	21 [212]	30 [256]	22 [212]	20 [256]
		p = 0.63	p = 0.49	p = 0.004	p = 0.0002
**Pharynx**	wt	37 [286]	38 [402]	13 [286]	12 [402]
	35 del	44 [298]	42 [508]	24 [298]	17 [508]
		p = 0.08	p = 0.2	p = 0.003	p = 0.04
**Palate**	wt	33 [374]	33 [300]	12 [374]	15 [300]
	35 del	34 [592]	31 [390]	17 [592]	18 [390]
		p = 0.73	p = 0.88	p = 0.04	p = 0.3
**Telencephalon**	wt	31 [504]	32 [520]	14 [504]	15 [520]
	35 del	32 [460]	36 [480]	19 [460]	21 [480]
		p = 0.84	p = 0.2	p = 0.03	p = 0.009
**Diencephalon**	wt	29 [400]	31 [460]	6 [400]	8 [460]
**Floorplate**	wt	34 [216]	35 [208]	-	-
	35 del	32 [242]	38 [262]		
		p = 0.69	p = 0.5		
**Pre-motor neurons**	wt	-	-	23 [186]	10 [156]
	35 del			18 [186]	16 [176]
				p = 0.37	p = 0.1

Statistical analysis of data for Fig. 1E & Supplemental Fig S1A, F. Table shows the percent of alleles transcribing at *Shh* and *Mnx1* as assayed by RNA FISH signal in wild type (wt) and 35 kb deletion (35 del) E11.5 mouse embryos in tissues of the floorplate, pre-motor neurons ZPA, pharynx, palate, telencephalon and diencephalon. Number of alleles scored is indicated in square brackets. *p* values are from two-sided Fisher's exact test comparing data from wild-type and 35 del embryos.

Table S2. Significance of the frequency of transcription at *Mnx1* or *Shh* activated by enhancers of the other gene

E10.5 *Shh*-exp tissue (*Shh* Non-exp [ ])	Distance Shh enhancer to *Mnx1* (kb)	Transcribing alleles (%)	
		Mnx1	***Shh***
**[Dorsal neural tube]**	N/A	2	2
**[adj. ZPA]**	N/A	3	1
**[Hb/nt dorsal neural tube]**	N/A	3	1
**[adj. Brain]**	N/A	2	5
**[Pre-motor neurons]**	N/A	25	5 ($p = 0.41$)
**Floorplate**	>1000kb	5 ($p = 0.14$)	52
**Hb/nt floorplate**	900kb	4 ($p = 0.77$)	54
**Diencephalon**	600kb	8 ($p = 0.002$)	31
**Telencephalon**	580kb	15 ($p < 0.0001$)	32
**Palate**	350kb	15 ($p < 0.0001$	33
**Pharynx**	280kb	12 ($p < 0.0001$)	38
**Foregut**	250kb	15 ($p < 0.0001$)	40
**Lung buds**	250kb	17 ($p < 0.0001$)	43
**ZPA**	150kb	20 (p < 0.0001)	37

Statistical analysis of data for Supplemental Fig. S1B, C & F. Comparison of *Mnx1* transcription in *Shh*-expressing tissues of E10.5 embryos, regulated by either *Shh* proximal or distal enhancers, and comparison of transcription of both genes in non-expressing tissue with tissue expressing the other gene. *Shh* and *Mnx1* non-expressing tissues: dorsal neural tube, limb bud adjacent to the ZPA and head tissue adjacent to the brain. *Mnx1*-expressing tissue (to identify significant *Shh* activation by *Mnx1* enhancers): pre-motor neurons (proximal *Mnx1* enhancers). *Shh*-expressing tissues: floorplate (proximal *Shh* enhancers), and ZPA, foregut, lung buds, pharynx, palate, telencephalon, diencephalon, and hindbrain/neural tube (floorplate) (distal *Shh* enhancers). The distances (kb) between Mnx1 and the relevant Shh enhancer is shown. N/A = not applicable. *p*-values from Fisher's Exact Tests.

Table S3. Comparison of co-transcription at *Shh* and *Mnx1* in wild type and 35kb deletion ZPA and mESCs

Tissues/cells	Genotype	Shh & *Mnx1* co-transcribing alleles (%)	
		Replicate 1	Replicate 2
**E11.5 ZPA**	wt<br>35 del	23<br>44 ($p = 0.02$)	12<br>35 ($p = 0.0004$)
**E11.5 pharynx**	wt<br>35 del	17<br>39 ($p < 0.0001$)	13<br>11 ($p = 0.56$)
**E11.5 palate**	wt<br>35 del	22<br>13 ($p = 0.047$)	21<br>20 ($p = 0.88$)
**E11.5 telencephalon**	wt<br>35 del	19<br>31 ($p = 0.01$)	21<br>14 ($p = 0.06$)
**E14 mESCs (tZRS-Vp64)**	wt<br>35 del	39<br>35 ($p = 0.63$)	28<br>32 ($p = 0.69$)

Statistical analysis of data for Fig. 2B,E & Supplemental Fig. S2A,C. Table shows the percent of alleles transcribing at *Shh* and *Mnx1* as assayed by RNA-FISH signal in wild type (wt) and 35 kb deletion (35 del) in the ZPA, pharynx, palate and telencephalon of E11.5 mouse embryos and in mESCs transfected with tZRS-Vp64. Values are proportion of total transcribed alleles. *p*-values from Fisher's Exact Tests.

Table S4. Comparison of the frequency of concurrent transcription at *Shh* and *Mnx1* in cis vs in trans

Tissue/cell type	Genotype	Number of nuclei	Model coefficient estimate	*p* value	Significance (*p* < 0.05)
**E10.5**					
**Foregut**	wt	37	0.86	0.017	True
**Lung bud**	wt	43	-0.05	0.88	False
**ZPA**	wt	149	0.07	0.68	False
**E11.5**					
**ZPA**	wt	20	1.73	0.0056	True
	35 del	54	3.97	0.0001	True
**Pharynx**	wt	26	0.64	0.12	False
	35 del	98	1.63	< 0.0001	True
**Palate**	wt	58	1.69	< 0.0001	True
	35 del	89	0.94	0.0001	True
**Telencephalon**	wt	91	1.4	< 0.0001	True
	35 del	97	1.41	< 0.0001	True
**Diencephalon**	wt	49	1.49	0.0001	True
**E14 mESCs**	wt	33	1.5	0.0009	True
**(tZRS-Vp64)**	35 del	30	0.55	0.15	False

Data associated with Fig. 2B,E & Supplemental Fig. S2A,C and F. Analysis in E10.5 tissues from wild-type (wt) animals and in E11.5 tissue and E14 mESCs for both wt and 35kb del genotypes. E14 mESCs were transfected with tZRS-Vp64. Only nuclei containing one *Shh* and one *Mnx1* signal were analyzed using logistic regression, a form of fitted generalized linear regression model, to determine if there were more nuclei where both genes are being transcribed on the same chromosome (in cis) or where they are being transcribed on separate chromosomes. A coefficient estimate value close to 0 indicates equal numbers of co-transcribed and individually transcribed alleles, a positive value indicates preferential co-transcription and a negative value indicates preferential individual transcription. *P*-values from z-Test.

Table S5. Proportion of *Shh* and *Mnx1* transcribing alleles in E14 wild type and 35kb deletion mESCs transfected with either tZRS-Vp64 or tZRS-Δ

E14 genotype	Tranfection	Transcribing alleles (%) Number of alleles [ ]			
		Shh		*Mnx1*	
		Replicate 1	Replicate 2	Replicate 1	Replicate 2
**Wild type**	tZRS-Vp64	16 [392]	22 [186]	13 [392]	18 [186]
	ZRS-Δ	3 [132] $p = 0.0004$	7 [106] $p = 0.0008$	3 [132] $p = 0.0005$	6 [106] $p = 0.004$
**35 del**	tZRS-Vp64	20 [250] $p = 0.14$	25 [140] $p = 0.51$	23 [250] $p = 0.002$	28 [140] $p = 0.03$
	ZRS-Δ	7 [200] $p < 0.0001$	7 [92] $p = 0.0002$	7 [200] $p < 0.0001$	7 [92] $p < 0.0001$

Statistical analysis of data for Fig. 2D & Supplemental Fig. S2B. *p*-values from Fisher's Exact Tests. tZRS-Δ values were compared with those from tZRS-Vp64 for the same cell type. 35 del cells transfected with tZRS-Vp64 were compared with wild type cells transfected with tZRS-Vp64.

Table S6. Distribution of *Shh*-ZRS, *Mnx1*-ZRS, *Shh-Mnx1* interprobe distances in ZPA cells at non-, *Shh*-, *Mnx1*- and *Mnx1* & *Shh*-transcribing alleles

Data for	Tissue	Transcription	No. alleles	***Shh*-ZRS**	***Mnx1*-ZRS**	***Shh-Mnx1***
				**Median & *inter-quartiles* (nm) Number of alleles [ ]**		
**Fig. 3B, Supplemental Fig. S3B**	**ZPA** (rep1)	**none**	120	332, *247/614*	565, *425/779*	545, *433/820*
		Shh	77	291, *184/379* ($p = 0.0007$)	477, *339/726* ($p = 0.18$)	518, *395*/721 ($p = 0.46$)
		Mnx1	20	270, *238/450* ($p = 0.23$)	262, *206/378* ($p < 0.0001$)	356, *279/526* ($p = 0.0009$)
		Shh* & *Mnx1	21	274, *210/307* ($p = 0.05$)	294, *274/405* ($p < 0.0001$)	376, *238/463* ($p < 0.0001$)
	**ZPA** (rep2)	**none**	100	272, *234/372*	442, *264/566*	463, *394/636*
		Shh	38	283, *229/324* ($p = 0.8$)	533, *324/696* ($p = 0.03$)	622, *461/742* ($p = 0.03$)
		Mnx1	11	321, *260/407* ($p = 0.46$)	256, *111/345* ($p = 0.002$)	301, *279/405* ($p = 0.006$)
		Shh* & *Mnx1	41	238, *206/294* ($p = 0.007$)	264, *206/445* ($p = 0.0002$)	347, *264/486* ($p = 0.0002$)
**Fig. 3E, Supplemental Fig. S3D**	**pmn** (rep1)	***Mnx1***	27	379, *258/478*	541, *354/938*	506, *369/890*
		**none**	57	333, *271/463* ($p = 0.63$)	520, *420/813* ($p = 0.82$)	584, *420/740* ($p = 0.67$)
	**ZPA** (rep1)	***Mnx1***	41	274, *210/350* ($p = 0.07$)	288, *233/392* ($p < 0.0001$)	376, *247/491* ($p = 0.005$)
		**none**	197	322, *238/471* ($p = 0.24$)	551, *385/762* ($p < 0.0001$)	527, *420/773* ($p < 0.0001$)
	**pmn** (rep2)	***Mnx1***	32	365, *316/479*	618, *388/922*	734, *507/1035*
		**none**	38	394, *321/514* ($p = 0.5$)	579, *359/794* ($p = 0.59$)	694, *511/798* ($p = 0.45$)
	**ZPA** (rep2)	***Mnx1***	52	247, *223/306* ($p < 0.0001$)	264, *201/405* ($p < 0.0001$)	321, *264/469* ($p < 0.0001$)
		**none**	138	278, *229/350* ($p = 0.047$)	459, *268/673* ($p < 0.0001$)	494, *398/690* ($p < 0.0001$)

Statistical analysis of DNA-FISH *Shh-ZRS*, *Mnx1-ZRS* and *Shh-Mnx1* inter-probe distance data for Fig. 3B, E. and Supplemental Fig. S3B, D. Data are from two biological replicates with Number of alleles scored shown. Median distances (nm) are shown together with inter-quartile distances in italics. p-values from Mann-Whitney U Tests. For Fig. 3B/ Fig. S3B data p values are in comparison to alleles not transcribing either *Shh* or *Mnx1*. For Fig3E/ Fig. S3D data p values *Mnx1*-transcribing and non-transcribing values were compared for each tissue (ZPA or pre-motor neuron (pmn)) and *Mnx1*-transcribing values in pmn tissues were compared with ZPA *Mnx1*-transcribing values.

Table S7. Categorical analysis of the spatial relationship of *Shh*, ZRS and *Mnx1* in ZPA

Data for	Tissue	Transcription	*Shh*-ZRS	*Mnx1*-ZRS	*Shh-Mnx1*
			**Alleles < 350nm apart (%)**		
**Fig. 3C**	**ZPA**	**none**	60	24	18
		Shh	74 (p = 0.02)	26 (p = 0.67)	22 (p = 0.45)
		Mnx1	68 (p = 0.44)	71 (p < 0.0001)	58 (p < 0.0001)
		Shh* & *Mnx1	84 (p = 0.001)	64 (p < 0.0001)	49 (p < 0.0001)
**Fig. 3F**	**PMN**	***Mnx1***	47	19	13
		**None**	44 (p = 0.74)	20 (p = 0.84)	19 (p = 0.36)
	**ZPA**	***Mnx1***	78 (p < 0.0001)	66 (p < 0.0001)	52 (p < 0.0001)
		**None**	66 (p = *0.03*)	25 (p < *0.0001*)	20 (p < *0.0001*)

Categorical analysis of the inter-probe distances between *Shh*-ZRS, *Mnx1-ZRS* and *Shh-Mnx1* in ZPA and PMN cells at non-, *Shh*-, *Mnx1*- and *Shh* & *Mnx1*-transcribing alleles (Fig. 3C) and at non- and *Mnx1*-transcribing alleles (Fig. 3F). Statistical analysis of data for Figs. 3C & F with replicate data sets combined. *p*-values from Fisher's Exact Tests are for comparison to non-expressing alleles for Fig. 3C data, and to *Mnx1* expressing alleles for data in Fig. 3F.

Table S8. Proportion of *Shh* and *Mnx1* transcribing alleles in E14 CTCF-AID and SCC1-AID - or + auxin mESCs transfected with tZRS-Vp64

E14 mESCs	Transcribing alleles (%)<br>Number of alleles [ ]			
	Shh		*Mnx1*	
	Rep 1	Rep 2	Rep 1	Rep 2
**CTCF-AID**				
**- auxin**	22 [238]	22 [134]	18	16
**+ auxin**	16 [300]	19 [132]	15	15
	(p = 0.12)	(p = 0.55)	(p = 0.29)	(p = 0.87)
**SCC1-AID**				
**- auxin**	20 [200]	19 [218]	17	17
**+ auxin**	7 [290]	7 [254]	10	11
	(p < 0.0001)	(p < 0.0001)	(p = 0.03)	(p = 0.04)

Statistical analysis of data for Figs. 4A & Supplemental Fig. S4A examining the consequence of CTCF or SCC1 ablation (using auxin induced degradation) on the proportion of alleles transcribing at *Shh* or *Mnx1* in mESCs transfected with tZRS-Vp64. *p*-values from Fisher's Exact Tests compare – vs + auxin data for two biological replicates. Number of alleles scored indicated in square brackets.

Table S9. Distribution of distances between *Shh*-ZRS, *Mnx1*-ZRS, *Shh-Mnx1* DNA-FISH signals in E14 SCC1-AID - or + auxin mESCs transfected with tZRS-Vp64

SCC1-AID	No. of alleles	Median & *inter-quartiles* (nm)		
		Shh-ZRS	*Mnx1*-ZRS	*Shh-Mnx1*
Rep1				
**- auxin**	78	345, *258/520*	255, *133/300*	411, *295/616*
**+ auxin**	90	542, *351/786*	455, *238/485*	476, *323/720*
		($p < 0.0001$)	($p < 0.0001$)	($p = 0.05$)
Rep2				
**- auxin**	87	449, *288/514*	300, *229/461*	485, *334/668*
**+ auxin**	75	603, *419/857*	481, *259/710*	551, *370/861*
		($p < 0.0001$)	($p = 0.0005$)	($p = 0.09$)

Statistical analysis of DNA-FISH *Shh-ZRS*, *Mnx1-ZRS* and *Shh-Mnx1* inter-probe distance data for Fig. 4C. and Supplemental Fig. S4B,C. Data are from two biological replicates with Number of alleles scored are indicated. Median distances (nm) are shown together with inter-quartile distances in italics. *p*-values from Mann-Whitney U Tests comparison data for – vs + auxin.

Table S10. Categorical analysis of the spatial relationship of *Shh*, ZRS and *Mnx1* in E14 SCC1-AID - & + auxin mESCs

SCC1-AID	Alleles < 350nm apart (%)		
	***Shh*-ZRS**	***Mnx1*-ZRS**	***Shh-Mnx1***
**-auxin**	44	70	32
**+auxin**	21 (p < 0.0001)	42 (p < 0.0001)	28 (p = 0.47)

Statistical analysis of data for Figs. 4C & C. *p*-values from Fisher's Exact Tests compare data for - vs + auxin..

Table S11. Fosmid Probes for DNA FISH

Region	Whitehead (Sanger) Name	Ensembl name	Coordinates Start	Coordinates End	Size (bp)
Shh	WI1-0574O18	G135P64333A4	28754458	28795879	41421
ZRS	WI1-1047E14	G135P600929F6	29611727	29653695	41968
Mnx1	WI1-1204B6		29791124	29827491	36368

Names are Ensembl (r 45) (http://jun2007.archive.ensembl.org/Mus_musculus/index.html).
Mouse genome assembly number: NCBI m37.
